# Quantitative methods used to evaluate impact of health promotion interventions to prevent HIV infections: a methodological systematic review protocol

**DOI:** 10.1186/s13643-022-01970-z

**Published:** 2022-05-06

**Authors:** Andrainolo Ravalihasy, Lidia Kardaś-Słoma, Yazdan Yazdanpanah, Valéry Ridde

**Affiliations:** 1 Centre Population et Développement (IRD, Université Paris Cité, Inserm ERL 1244 SAGESUD), 75006 Paris, France; 2 Institut de recherche pour le développement, Marseille, France; 3 French Collaborative Institute on Migrations, CNRS, Aubervilliers, France; 4 UMR 1137, Inserm, University of Paris, IAME, Paris, France

**Keywords:** Evidence-based impact evaluation, Combination HIV prevention, Health promotion, Study design, Statistical methods, Mathematical methods

## Abstract

**Background:**

Combination prevention is currently considered the best approach to combat HIV epidemic. It is based upon the combination of structural, behavioral, and biomedical interventions. Such interventions are frequently implemented in a health-promoting manner due to their aims, the approach that was adopted, and their complexity. The impact evaluation of these interventions often relies on methods inherited from the biomedical field. However, these methods have limitations and should be adapted to be relevant for these complex interventions. This systematic review aims to map the evidence-based methods used to quantify the impact of these interventions and analyze how these methods are implemented.

**Methods:**

Three databases (Web of Science, Scopus, PubMed) will be used to identify impact evaluation studies of health promotion interventions that aimed at reducing the incidence or prevalence of HIV infection. Only studies based on quantitative design assessing intervention impact on HIV prevalence or incidence will be included. Two reviewers will independently screen studies based on titles and abstracts and then on the full text. The information about study characteristics will be extracted to understand the context in which the interventions are implemented. The information specific to quantitative methods of impact evaluation will be extracted using items from the Mixed Methods Appraisal Tool (MMAT), the guidelines for reporting Statistical Analyses and Methods in the Published Literature (SAMPL), and the guidelines for Strengthening The Reporting of Empirical Simulation Studies (STRESS). This review will be conducted according to the Preferred Reporting Items for Systematic Review and Meta-Analyses (PRISMA) statement.

**Discussion:**

The impact evaluation of HIV prevention interventions is a matter of substantial importance given the growing need for evidence of the effectiveness of these interventions, whereas they are increasingly complex. These evaluations allow to identify the most effective strategies to be implemented to fight the epidemic. It is therefore relevant to map the methods to better implement them and adapt them according to the type of intervention to be evaluated.

**Systematic review registration:**

PROSPERO CRD42020210825

**Supplementary Information:**

The online version contains supplementary material available at 10.1186/s13643-022-01970-z.

## Background

The importance of health promotion interventions is acknowledged, thanks to their impacts on social determinants of health and the need to fight against health inequities [1, 2]. Despite this recognition, their effectiveness is often debated and criticized due to the lack of available evidence from impact evaluations using quantitative data, especially randomized controlled trials. However, these methods, inherited from the biomedical field, have several methodological and practical limitations for quantifying the impact of this type of intervention and should be adapted to be relevant [3, 4].

In the context of the fight against HIV epidemic, the lack of curative treatment has brought health promotion interventions at the forefront, particularly those aimed at preventing the transmission of the virus. Currently, biomedical interventions alone have shown their limits, and combination prevention is considered the best approach to curb the epidemic [5, 6]. It is based upon the combination of structural, behavioral, and biomedical interventions that address specific prevention needs at the individual and community levels. The interventions which fall within this framework are frequently implemented in a health-promoting manner due to their aims, the approach adopted, and their complexity. Over the past several years, such interventions have been proposed and evaluated for effectiveness in a variety of ways. Among those evaluated using evidence-based quantitative methods, some have been shown to be effective, while others have not. The challenge of impact evaluation of these interventions is paramount in helping to identify and decide which programs and policies should be implemented and supported to address the epidemic. It is essential that these evaluations are conducted in a rigorous manner which is appropriate to the nature and the complexity of health promotion interventions. Hence, we decided to conduct a review to identify and assess the implementation of the evidence-based impact of evaluation methods that are used to assess these interventions.

### Objectives

This review is conducted to systematically review quantitative methods for evaluating the impact of interventions that aimed at preventing HIV transmission during sexual exposure. The specific questions this review attempts to answer are as follows:

- What quantitative methods (statistical or mathematical) are used to quantify intervention impact on HIV incidence or prevalence?
- What are the designs of these studies?
- Are quantitative methods implemented appropriately?

Answering these questions will allow a critical synthesis of the methods used to assess the impact of the interventions concerned by this systematic review and more broadly to identify directions for methodological development to adapt these methods when assessing the impact of interventions designed to address social and behavioral determinants of health.

## Methods/design

This systematic review is developed in accordance with the Preferred Reporting Items for Systematic Reviews and Meta-Analyses Protocols (PRISMA-P) statement [7] (Additional file 1). The protocol has been registered in the International Prospective Register of Systematic Reviews (PROSPERO): CRD42020210825.

### Eligibility criteria

This review focuses on impact evaluation studies of health promotion interventions designed to prevent HIV transmission during sexual risk exposure. Health promotion is defined as a process of enabling individuals to improve their health and their control over their health [8]. For this reason, the studies to be included involve behavioral and/or structural components that may or may not be supplemented by the use of biomedical prevention tools. More specifically, this review will include studies based solely on existing interventions; studies based on hypothetical interventions and exclusively simulated data will be excluded. Hence, modeling studies may be included as long as the model inputs are based on data from an existing intervention to be evaluated. The outcomes studied in this review are quantitative methods based on strictly quantitative designs (randomized controlled intervention studies, nonrandomized controlled intervention studies, observational studies) that meet the classification proposed in Deeks et al. [9] where controlled studies are those where the intervention assignment is done by researchers. Studies that do not meet these conditions will be excluded. Finally, the review focuses on interventions designed to reduce HIV transmission; eligible studies are those that assess intervention impact on HIV prevalence or incidence as a primary or secondary outcome.

### Literature search

Five databases were considered in order to identify the studies to be included in the review: Web of Science, Scopus, PubMed, CINAHL, and PsycINFO. Finally, a sensitivity analysis of the search equation showed that three databases (Web of Science, Scopus, PubMed) were sufficient to uncover the studies relevant to this review. Moreover, it has been shown that the joint use of these databases allows to ensure an adequate literature search performance [10]. The search strategy consisted of using three groups of terms and two filters:

i) Terms associated with HIV prevalence and incidence: to restrict the results to studies that aim to reduce HIV transmission
ii) Terms associated with the notion of impact of interventions: to focus the search on articles referring to intervention impact evaluation
iii) Terms associated with prevention interventions and sexual risk exposure: to focus the search on interventions designed to reduce the occurrence of new HIV cases and to focus the search on interventions targeting behavioral and structural barriers.
iv) Restricting the search to articles written in English and French
v) Restricting the search to scientific articles and not retracted publications

The search strategy is reported in Table 1 and was developed and tailored to each database with the assistance of a trained librarian.

**Table 1:** Search strategy

Databases	Search terms
**Web of Science**	i) **Terms associated with prevalence and incidence of HIV infection** TS=(HIV OR "HIV infection" OR "human immunodeficiency virus") AND TS=(prevalence OR incidence)
	ii) **Terms associated with the notion of effectiveness of interventions** AND TS=(effect OR effects OR efficacy OR effectiveness OR impact*) AND TS=(intervention* OR program* OR project OR trial*)
	iii) **Terms associated with prevention interventions and sexual risk exposure** AND TS=(prevention OR prophylaxis OR "primary prevention") AND TS=("unsafe sex" OR "safe sex" OR "sex work" OR "sex worker" OR "sex workers" OR "unprotected sex" OR "protected sex" OR "high-risk sex" OR "high risk sex" OR "sexual risk")
	iv) **Language** AND LA=(English OR French)
	v) **Document type** NOT DT=("RETRACTED PUBLICATION" OR BOOK OR "book chapter" OR "PROCEEDINGS PAPER" OR "MEETING ABSTRACT")
**PubMed**	i) **Terms associated with prevalence and incidence of HIV infection** (HIV [Title/Abstract] OR "HIV infection"[Title/Abstract] OR "human immunodeficiency virus"[Title/Abstract]) AND (prevalence[Title/Abstract] OR incidence[Title/Abstract])
	ii) **Terms associated with the notion of effectiveness of interventions** AND (effect [Title/Abstract] OR effects [Title/Abstract] OR efficacy [Title/Abstract] OR effectiveness [Title/Abstract] OR impact*[Title/Abstract]) AND (intervention*[Title/Abstract] OR program*[Title/Abstract] OR project[Title/Abstract] OR trial*[Title/Abstract])
	iii) **Terms associated with prevention interventions and sexual risk exposure** AND (prevention [Title/Abstract] OR prophylaxis[Title/Abstract] OR "primary prevention"[MeSH]) AND ("unsafe sex"[MeSH] OR "safe sex"[MeSH] OR "sex work"[MeSH] OR "unprotected sex"[Title/Abstract] OR "protected sex"[Title/Abstract] OR "sexual risk"[Title/Abstract] OR "high-risk sex"[Title/Abstract] OR "high risk sex"[Title/Abstract])
	iv) **Language** AND (english[Filter] OR french[Filter])
	v) **Document type** NOT (booksdocs[Filter] OR congress[Filter] OR retractedpublication[Filter])
**Scopus**	i) **Terms associated with prevalence and incidence of HIV infection** TITLE-ABS-KEY(hiv OR "HIV infection" OR "human immunodeficiency virus") AND TITLE-ABS-KEY (prevalence OR incidence)
	ii) **Terms associated with the notion of effectiveness of interventions** AND TITLE-ABS-KEY(effect OR effects OR efficacy OR effectiveness OR impact*) AND TITLE-ABS-KEY (intervention* OR program* OR project OR trial*)
	iii) **Terms associated with prevention interventions and sexual risk exposure** AND TITLE-ABS-KEY ( prevention OR prophylaxis OR "primary prevention") AND TITLE-ABS-KEY("unsafe sex" OR "safe sex" OR "sex work" OR "sex worker" OR "sex workers" OR "unprotected sex" OR "protected sex" OR "high-risk sex" OR "high risk sex" OR "sexual risk")
	iv) **Language** AND (LIMIT-TO (LANGUAGE, "English") OR LIMIT-TO (LANGUAGE, "French"))
	v) **Document type** AND (EXCLUDE(DOCTYPE, "tb") OR EXCLUDE(DOCTYPE, "ch") OR EXCLUDE(DOCTYPE, "cp")

### Data collection

#### Study selection

References management will be done using a bibliographic management software (Zotero 5.0.96.2). Screening and data extraction will be done using a systematic review management software (Covidence). Duplicates will be removed automatically (using Covidence) and also manually. The study selection will be done in two steps: (i) a selection based on titles and abstracts and (ii) a selection based on full texts. At each stage of the selection, two reviewers will independently select the studies taking into account the eligibility criteria. At the end of each stage, disagreements on which studies to include will be resolved with the help of a third senior reviewer. Only peer-reviewed scientific articles will be considered.

#### Data extraction

Two types of data will be extracted from the studies: general information about the included studies and specific information about the methods used to evaluate interventions.

##### *Data concerning the characteristics of the studies*

These information will help to describe the included studies and the contexts in which the interventions are implemented. These information concern the authors, the title of the article, the date of publication, the place where the studies were carried out, the purpose, and the results of the studies.

##### *Data on impact evaluation methods*

A data extraction grid was developed for the purposes of this review (Table 2). This grid has three sections that allow for the extraction of (i) information about the design of the studies, (ii) information about the statistical methods (when appropriate), and (iii) information about the mathematical methods (when appropriate). The items used in the extraction grid are based on tools and recommendations consistent with each of the abovementioned sections and with the investigated research question in the review [11–13]. We also verified that the information to be extracted is consistent with the standards of reporting according to the study design, the CONSORT statement and the relevant extensions for randomized controlled studies [14–17], the TREND statement for non-randomized controlled studies [18], and the STROBE statement for observational studies [19].

**Table 2:** Quality assessment grid

Section	Items
**Study design informations**	**Randomized controlled trials**
	Is randomization appropriately performed?
	Are the groups comparable at baseline?
	Are there complete outcome data?
	Are outcome assessors blinded to the intervention provided?
	Did the participants adhere to the assigned intervention?
	**Non-randomized controlled trials/observational studies**
	Are the participants representative of the target population?
	Are measurements appropriate regarding both the outcome and intervention (or exposure)?
	Are there complete outcome data?
	Are the confounders accounted for in the design and analysis?
	During the study period, is the intervention administered (or exposure occurred) as intended?
**Information about statistical methods**	Is the minimum difference considered important to prove the intervention efficacy/effectiveness reported?
	Are the alpha and beta levels that define statistical significance reported and respected?
	What is the statistical method used to assess the intervention impact on the primary outcome?
	What is statistical the method used to assess the intervention impact on HIV prevalence/incidence?
	Did the authors report analyses to confirm that the statistical assumptions of the analysis were met or the goodness of fit of the model?
	Did the authors report a measure of precision alongside the impact measure?
	Is the design taken into account in the statistical analyses?
**Information about the mathematical methods**	Are the parameters to be estimated in the model specified?
	Are the uncertainties concerning these parameters evaluated?
	Are the different components of the model and their roles clearly defined?
	Are the different scenarios/processes/assumptions tested by the model clearly specified?
	Are the data and data sources specified?
	Are the states of the various model components clearly specified from data?
	Are the methods used to compute estimates specified?

To test the applicability of the grid, a sample of 14 articles selected on the basis of their content was used (Additional file 2): 6 articles concerning randomized controlled studies (2 of which present mathematical impact evaluation), 3 articles concerning nonrandomized controlled studies, and 5 articles concerning observational studies (2 of which present mathematical impact evaluation). These articles were selected independently of the research questions addressed in the review to be conducted. The item development for each section of the extraction grid is presented below.

##### Study design information

Eligible studies are based on strictly quantitative designs, i.e., randomized controlled studies, non-randomized controlled studies, or observational studies. Items to extract study design-specific information originate from the Mixed Methods Appraisal Tool (MMAT). This tool allows to assess the design quality of quantitative studies [12, 20, 21]. Because these questions are specific to study design, the information to be extracted is different according to the study type. These items offer a choice of three possible answers (yes, no, unclear).

##### Information about statistical methods

Items concerning statistical methods are formulated using the guidelines for reporting Statistical Analyses and Methods in the Published Literature (SAMPL) [11]. The criteria identified in these guidelines are related to the implementation of statistical methods. The developed items allow the extraction of information common to different statistical methods for purpose to compare included studies. These items can be categorized into two types: (i) those that answer questions about the sample size and (ii) those that answer questions about the implementation of statistical methods in the analyses. The formulated items offer a choice of four possible answers (yes, no, imprecise, not concerned) except for one item allowing the classification of statistical methods (seven categories: correlation analysis, regression analysis, ANOVA/ANCOVA, bayesian analyses, statistical tests, other, not concerned).

##### Information about mathematical methods

Items concerning mathematical methods are formulated using the guidelines for Strengthening The Reporting of Empirical Simulation Studies (STRESS) [13]. Several criteria identified from these guidelines allow the collection of information which is consistent with several types of models. The developed items are based on these criteria and can be categorized into four types: (i) description of the objectives of the model, (ii) description of the assumptions made by the model, (iii) description of the data used in the model, and (iv) description of the implementation of the model. These items offer a choice of four possible answers (yes, no, unclear, not concerned).

### Data analysis

This review will be conducted in accordance with the Preferred Reporting Items for Systematic Reviews and Meta-Analyses (PRISMA) statement [22]. The data synthesis will report all applicable items from this checklist. This synthesis will present four parts concerning the general characteristics of the included studies and the three sections of the developed extraction grid. The first part, which deals with general information about the studies, provides a summary of the context, the scope of the studies, the assumptions made, and the presented results. The second part, concerning the study design, gives insights about the methodological quality of the considered studies. The third and fourth sections allow to identify and assess the implementation of quantitative methods. This synthesis will be presented using descriptive tables and graphs and interpreted accordingly. All analyses will be presented according to study design and regardless of the design when relevant.

## Discussion

Intervention impact evaluation is one of many tools that support evidence-based program evaluation. It allows to determine whether an intervention improves significantly the situation of those who benefit from it compared to those who do not in the context of the study. Henceforth, this type of evaluation has been rooted in evaluation practices for the purpose of quantifying intervention effects and has legitimated a form of hierarchy among methods in terms of evidence [23]. Yet, these methods (including randomized controlled trials) are not without limitations, and while useful, their implementation requires better understanding of what kind of evidence they provide as well as how they may be used to inform research and practices [24–26]. In addition to the general criticisms, these methods have specific limitations regarding the field of health promotion and should be tailored before being used [27–29].

In the context of the fight against HIV epidemic, there is an urgent demand for evidence-based health promotion interventions to make available enough prevention strategies to stem the epidemic [30]. This dual demand for health promotion interventions and evidence warrants this review to map the methods commonly used to evaluate the impact of these interventions. This review will not only help to identify these methods but will also discuss the operationalization of these methods and the adaptations that have been made or could be made. Therefore, it is not intended to question the relevance of these studies which attempt to evaluate and make available useful interventions. On the contrary, it responds to the need for knowledge about existing methods and to consider how to use them, improve them, and even design complementary tools to better evaluate interventions, especially in the field of health promotion.

This protocol has limitations. The included studies are expected to quantify the intervention impact on HIV transmission. Such studies rely on confirmatory methods based on quantitative data, thus leading to exclude mixed studies whose aims do not focus on hypothesis or theory testing [31]. Nonetheless, as long as the eligbility criteria are met and the data extraction grid remains applicable, such study will be considered. Similarly, all studies that do not refer to a measure of HIV prevalence or incidence as an outcome are not considered. Thus, we will not be able to identify all health promotion studies used to stem the epidemic. However, this choice is warranted given that the review focuses on quantitative methods, while the selected studies are supposed to assess the extent to which these interventions curb the epidemic.

Despite its limitations, this review will be useful in informing practices of impact evaluation of HIV programs. The current recommendations for evaluating HIV programs [32] acknowledge the relevance of numerous methods depending on the questions to be answered and the context of these programs. This review will help to identify the gap between these recommendations and what actually is being done, by identifying what methods are implemented and how they are implemented. In addition, the analysis of the implementation of the methods according to the study characteristics will allow to discuss in what circumstances each method applies. Thus, the results of this review will highlight some methodological challenges concerning the impact evaluation of complex interventions in order to guide future methodological developments. Given the need for evidence of the effectiveness of complex interventions, reviewing impact evaluation methods is necessary in order to map and improve these methods and consequently to improve decision-making pertaining to the concerned interventions.

## Supplementary information

**Additional file 1.** PRISMA-P 2015 Checklist.

**Additional file 2.** List of references used to test data extraction grid.

## Data Availability

All data relevant to the study are included in the article or uploaded as additional files.

## Abbreviations

CONSORT: Consolidated Standards of Reporting Trials
MMAT: Mixed Methods Appraisal Tool
PRISMA: Preferred Reporting Items for Systematic Reviews and Meta-Analyses
PRISMA-P: Preferred Reporting Items for Systematic Reviews and Meta-Analyses Protocols
SAMPL: Statistical Analyses and Methods in the Published Literature
STRESS: Strengthening The Reporting of Empirical Simulation Studies
STROBE: Strengthening the Reporting of Observational Studies in Epidemiology
TREND: Transparent Reporting of Evaluations with Nonrandomized Designs
