# Supplementary material for: Quantitative methods used to evaluate impact of health promotion interventions to prevent HIV infections: a methodological systematic review protocol
Source: Syst Rev. 2022 May 6;11:87. doi: 10.1186/s13643-022-01970-z (PMC9077925; doi:10.1186/s13643-022-01970-z)
Supplement: Supplementary file 2 — Additional file 2. List of references used to test data extraction grid. [file 13643_2022_1970_MOESM2_ESM.docx]

**Additional file 2**

**List of references used to test data extraction grid**

| **Design** | **References** |
| --- | --- |
| **Randomized controlled trials** | Eaton, L. A., Kalichman, S. C., Kenny, D. A., & Harel, O. (2013). A reanalysis of a behavioral intervention to prevent incident HIV infections : Including indirect effects in modeling outcomes of Project EXPLORE. AIDS Care, 25(7), 805‑811. https://doi.org/10.1080/09540121.2012.748870 |
|  | Jean, K., Boily, M.-C., Danel, C., Moh, R., Badjé, A., Desgrées-du-Loû, A., Eholié, S., Lert, F., Dray-Spira, R., Anglaret, X., & Ouattara, E. (2016). What Level of Risk Compensation Would Offset the Preventive Effect of Early Antiretroviral Therapy? Simulations From the TEMPRANO Trial. American Journal of Epidemiology, 184(10), 755‑760. https://doi.org/10.1093/aje/kww127 |
|  | Larke, N., Cleophas-Mazige, B., Plummer, M. L., Obasi, A. I. N., Rwakatare, M., Todd, J., Changalucha, J., Weiss, H. A., Hayes, R. J., & Ross, D. A. (2010). Impact of the MEMA kwa Vijana adolescent sexual and reproductive health interventions on use of health services by young people in rural Mwanza, Tanzania : Results of a cluster randomized trial. The Journal of Adolescent Health: Official Publication of the Society for Adolescent Medicine, 47(5), 512‑522. https://doi.org/10.1016/j.jadohealth.2010.03.020 |
|  | McCarthy, E., Joseph, J., Foster, G., Mangwiro, A.-Z., Mwapasa, V., Oyeledun, B., Phiri, S., Sam-Agudu, N. A., & Essajee, S. (2017). Modeling the Impact of Retention Interventions on Mother-to-Child Transmission of HIV : Results From INSPIRE Studies in Malawi, Nigeria, and Zimbabwe. Jaids-Journal of Acquired Immune Deficiency Syndromes, 75, S233‑S239. https://doi.org/10.1097/QAI.0000000000001364 |
|  | Özler, B., Hallman, K., Guimond, M.-F., Kelvin, E. A., Rogers, M., & Karnley, E. (2020). Girl Empower – A gender transformative mentoring and cash transfer intervention to promote adolescent wellbeing : Impact findings from a cluster-randomized controlled trial in Liberia. SSM - Population Health, 10, 100527. https://doi.org/10.1016/j.ssmph.2019.100527 |
|  | Sabin, L. L., Halim, N., Hamer, D. H., Simmons, E. M., Jonnalagadda, S., Larson Williams, A., Chemusto, H., Gifford, A. L., Bonawitz, R., Aroda, P., DeSilva, M., Gasuza, J., Mukasa, B., & Messersmith, L. J. (2020). Retention in HIV Care Among HIV-Seropositive Pregnant and Postpartum Women in Uganda : Results of a Randomized Controlled Trial. AIDS and Behavior. https://doi.org/10.1007/s10461-020-02875-5 |
| **Non randomized controlled trials** | Ameh, S., Klipstein-Grobusch, K., Musenge, E., Kahn, K., Tollman, S., & Gómez-Olivé, F. X. (2017). Effectiveness of an Integrated Approach to HIV and Hypertension Care in Rural South Africa : Controlled Interrupted Time-Series Analysis. JAIDS Journal of Acquired Immune Deficiency Syndromes, 75(4), 472‑479. https://doi.org/10.1097/QAI.0000000000001437 |
|  | Michielsen, K., Beauclair, R., Delva, W., Roelens, K., Van Rossem, R., & Temmerman, M. (2012). Effectiveness of a peer-led HIV prevention intervention in secondary schools in Rwanda : Results from a non-randomized controlled trial. Bmc Public Health, 12, 729. https://doi.org/10.1186/1471-2458-12-729 |
|  | Roux, P., Rojas Castro, D., Ndiaye, K., Debrus, M., Protopopescu, C., Le Gall, J.-M., Haas, A., Mora, M., Spire, B., Suzan-Monti, M., & Carrieri, P. (2016). Increased Uptake of HCV Testing through a Community-Based Educational Intervention in Difficult-to-Reach People Who Inject Drugs : Results from the ANRS-AERLI Study. PloS One, 11(6), e0157062. https://doi.org/10.1371/journal.pone.0157062 |
| **Observational studies** | Boily, M.-C., Pickles, M., Lowndes, C. M., Ramesh, B. M., Washington, R., Moses, S., Deering, K. N., Mitchell, K. M., Reza-Paul, S., Blanchard, J., Vassall, A., Alary, M., & Vickerman, P. (2013). Positive impact of a large-scale HIV prevention programme among female sex workers and clients in South India. AIDS, 27(9), 1449‑1460. https://doi.org/10.1097/QAD.0b013e32835fba81 |
|  | Jobarteh, K., Shiraishi, R. W., Malimane, I., Gudo, P. S., Decroo, T., Auld, A. F., Macome, V., & Couto, A. (2016). Community ART Support Groups in Mozambique : The Potential of Patients as Partners in Care. Plos One, 11(12), e0166444. https://doi.org/10.1371/journal.pone.0166444 |
|  | Kagaayi, J., Chang, L. W., Ssempijja, V., Grabowski, M. K., Ssekubugu, R., Nakigozi, G., Kigozi, G., Serwadda, D. M., Gray, R. H., Nalugoda, F., Sewankambo, N. K., Nelson, L., Mills, L. A., Kabatesi, D., Alamo, S., Kennedy, C. E., Tobian, A. A. R., Santelli, J. S., Ekstrom, A. M., … Reynolds, S. J. (2019). Impact of combination HIV interventions on HIV incidence in hyperendemic fishing communities in Uganda : A prospective cohort study. Lancet Hiv, 6(10), E680‑E687. https://doi.org/10.1016/S2352-3018(19)30190-0 |
|  | King, R., Min, J., Birungi, J., Nyonyintono, M., Muldoon, K. A., Khanakwa, S., Kaleebu, P., & Moore, D. M. (2015). Effect of Couples Counselling on Reported HIV Risk Behaviour among HIV Serodiscordant Couples by ART Use, HIV Status and Gender in Rural Uganda. Plos One, 10(9), e0136531. https://doi.org/10.1371/journal.pone.0136531 |
|  | Walker, J. G., Kuchuloria, T., Sergeenko, D., Fraser, H., Lim, A. G., Shadaker, S., Hagan, L., Gamkrelidze, A., Kvaratskhelia, V., Gvinjilia, L., Aladashvili, M., Asatiani, A., Baliashvili, D., Butsashvili, M., Chikovani, I., Khonelidze, I., Kirtadze, I., Kuniholm, M. H., Otiashvili, D., … Vickerman, P. (2020). Interim effect evaluation of the hepatitis C elimination programme in Georgia : A modelling study. Lancet Global Health, 8(2), E244‑E253. https://doi.org/10.1016/S2214-109X(19)30483-8 |
